# A comparison of bone conductivity on titanium screws inserted into the vertebra using different surface processing

**DOI:** 10.1186/s40634-020-00250-w

**Published:** 2020-05-13

**Authors:** Takashi Ota, Satoru Demura, Satoshi Kato, Katsuhito Yoshioka, Hiroyuki Hayashi, Kei Inoue, Kazuya Shinmura, Noriaki Yokogawa, Toshiharu Shirai, Hideki Murakami, Hiroyuki Tsuchiya

**Affiliations:** 1 Department of Orthopaedic Surgery, Graduate School of Medical Sciences, Kanazawa University, 13-1, Takara-machi, Kanazawa, 920-8641 Japan; 2 Department of Orthopaedics, Graduate School of Medical Science, Kyoto Prefectural University of Medicine, 465 Kajii-cho, Kawaramachi-Hirokoji, Kamigyo-ku, Kyoto, 602-8566 Japan; 3 Department of Orthopaedic Surgery, Nagoya City University Medical School, 1-Kawasumi, Mizuho-cho, Mizuho-ku, Nagoya, 467-8602 Japan

**Keywords:** Iodine-supported titanium implant, Osteoconductivity, Biomechanical analysis, Histological analysis

## Abstract

**Purpose:**

Antibacterial iodine-supported titanium has an anodized oxide layer; thus, it can be expected to have a higher osteoconductivity than untreated titanium. This study aimed to compare the osteoconductivity between untreated titanium (Ti), anodically oxidized titanium (AO-Ti), and iodine-supported titanium (I-Ti) screws.

**Methods:**

The screws were inserted into the vertebral bodies of 30 dogs (12 for the biomechanical, and 18 for the histological examination). The vertebral bodies were analyzed at 4 or 8 weeks after screw insertion. Biomechanically, rotational torque of the screw was measured. Histologically, bone formation index (ratio of the length of the part where the bone directly contacts with the length of the screw) and bone volume density (ratio of the area of the bone tissue to the area between the threads of the screw) were measured.

**Result:**

At 4 weeks, the torque value was significantly higher in the AO-Ti (0.59 ± 0.16 Nm) and I-Ti (0.72 ± 0.14 Nm) groups than in the Ti group (0.39 ± 0.12 Nm), with the AO-Ti and I-Ti groups showing no significant difference. Bone formation index was significantly higher in the AO-Ti (72.5% ± 0.8%) and I-Ti (73.4% ± 1.5%) groups than in the Ti group (64.6% ±1.7%), with the AO-Ti and I-Ti groups showing no significant difference. Bone volume density did not show a significant difference. At 8 weeks, the results were similar to those at 4 weeks.

**Conclusions:**

I-Ti had a higher osteoconductivity than Ti, indicating that iodine coating did not adversely affect osteoconductivity.

## Background

For the prevention of surgical site infection, innovative technologies such as silver coating, copper coating, gold coating, and fluorine coating on titanium implant surfaces has been attempted [2, 11, 14, 17]. In our institution, antibacterial iodine-supported titanium has been developed. One of the advantages is that iodine is considered to be safer than heavy metals, because it is physiologically used in the thyroid and excreted in the kidney [13, 16]. To make iodine-supported titanium, first, titanium is anodically oxidized so that the microporous structure appears on its surface, and second, the microporous structure is filled with povidone-iodine using special techniques [3].

According to the previous reports, anodically oxidized titanium showed excellent osteoconductivity on the metal surface [5, 7, 9, 10, 19]. Given that iodine-supported titanium also has an anodized oxide layer, it can be expected to have a higher bone conductivity than untreated titanium.

In the rabbit model, a study using a titanium cylindrical rod with a smooth surface compared the osteoconductivity between untreated titanium, anodically oxidized titanium, and iodine-supported titanium. In this study, anodic oxidized titanium and iodine-supported titanium showed a higher osteoconductivity than the untreated titanium histologically and biomechanically [15]. On the other hand, histological or biomechanical investigations of the screws having more complicated shapes than the cylindrical rods have not been conducted. Clinically, screws are frequently used in surgery; thus, investigation using screws is highly significant.

The purpose of the present study was to compare the osteoconductivity between untreated titanium, anodically oxidized titanium, and iodine-supported titanium screws inserted into the vertebral body biomechanically and histopathologically. In a previous report, iodine-supported titanium implants showed clinically excellent antimicrobial activity, osteoconductivity, and biocompatibility; thus, it was hypothesized that the osteoconductivity of I-Ti is higher than that of Ti and comparable to that of I-Ti [16].

## Methods

This study was designed for biomechanical and histological investigation by animal experiments. This study was conducted with approval from the Committee of Animal Care and Experimentation at Kanazawa University (Kanazawa, Japan, AP-163763). The animals used in this study were 1-year-old female beagle canines (body weight 10–12 kg) purchased from Japan SLC (Shizuoka, Japan). The dogs were housed separately in a stainless-steel cage, fed per the institutional animal care program feeding standard operating procedure, and provided with access to water ad libitum.

### Implant preparation

The implants used in this study were screws for spinal instrumentation with a length of 20 mm and a diameter of 3.5 mm (Vertex®, Medtronic Sofamor Danek, Memphis, TN, USA). The screw materials were untreated Ti-6Al-4 V titanium (Ti), Ti with anodic oxide layer on the surface (AO-Ti), and AO-Ti with iodine supported on the same anodic oxide layer (I-Ti). The iodine supports were produced by the Chiba Institute of Technology (Narashino, Japan) using a technique described by Hashimoto [3]. The thickness of the anodic oxide layer was between 5 and 10 μm with > 50,000 pores/mm^2^, which had the capacity to support 10–12 μg/cm^2^ of iodine. All screws were processed by Promedical Instruments Company (Kanazawa, Japan).

### Implantation

**Fig. 1:**
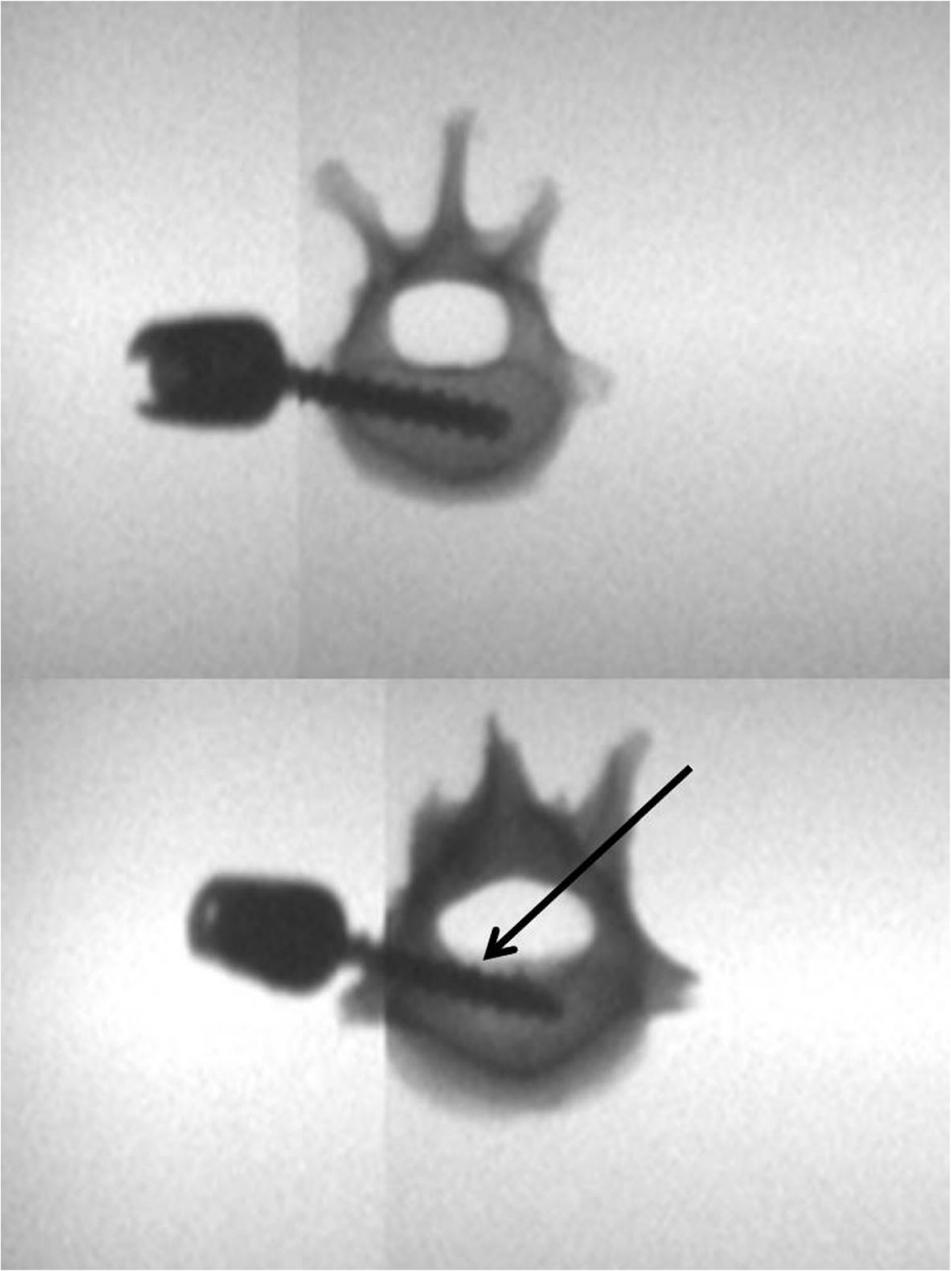
X-ray image of specimen. The insertion position of the screw was checked by an X-ray image. Specimens were adapted for examination if the screw was in contact with cancellous bone only (upper image), and excluded as an inappropriate position if any part of the screw was in contact with cortical bone (the arrow in the lower image)

Thirty female, 1-year-old beagle dogs were used in this study. Twelve dogs were used for the biomechanical examination, and 18 dogs were used for the histological examination. Dogs were anesthetized using an intravenous injection of propofol, inhalation anesthesia of nitrous oxide, and intramuscular injection of carprofen. All surgery was performed by the same surgeon (first author). A longitudinal skin incision was made in the middle of the back, and the fascia and paravertebral muscle were carefully retracted to expose the vertebral body. Screws with a length of 20 mm and a diameter of 3.5 mm were inserted directly into the vertebral bodies. Screws were inserted into L1 to L6 vertebral bodies (one screw was inserted into each vertebral body). With reference to previous reports, the vertebral bodies were removed at 4 or 8 weeks after implantation [10]. After propofol (3 mg/kg) was intravenously administered to sedate the animal, 20 mL of 1 M potassium chloride was administered intravenously for sacrifice. The insertion position of the screw was checked on the X-ray image. The specimens were examined if the screw was in contact with the cancellous bone only, and excluded if the screw was in an inappropriate position, i.e. any part of the screw was in contact with the cortical bone (Fig. 1).

### Biomechanical examination

**Fig. 2:**
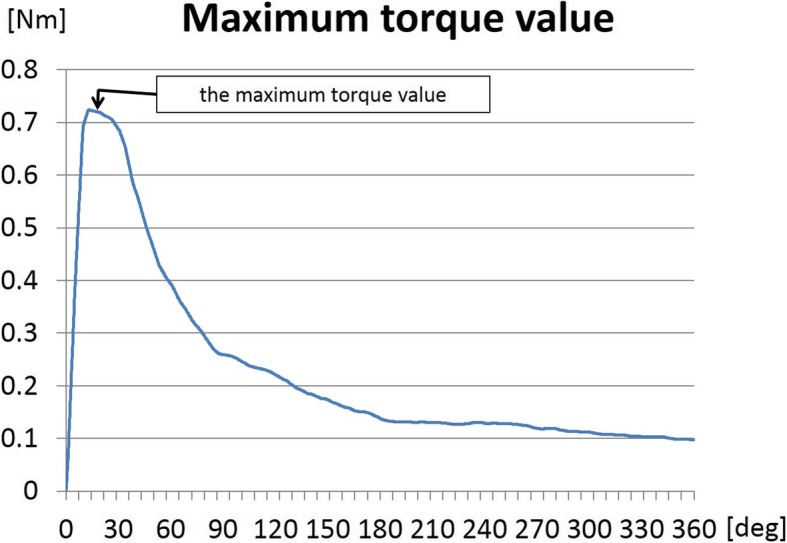
Maximum torque value. The torque value was measured continuously. The torque value reached the maximum at the moment when the screw began to rotate, and the value at that time was defined as the maximum torque value

Each vertebral body for biomechanical examination was fixed in polyethylene resin. The screw was fixed to a screwdriver and attached longitudinally to the load cell. Then, the torque value of the screw was measured using the torque meter MAX-T200NM (JAPAN INSTRUMENTATION SYSTEM CO., Ltd.). The torque examination was performed at the rate of 60°/sec until the screw turned 360° (6 s). The peak of the torque during the examination was adopted as the maximum torque value (Fig. 2) [1].

### Histological examination

The removed vertebral bodies were fixed with 10% formalin solution and 70% ethanol at room temperature. After fixation, the samples were fixed in methyl methacrylate resin, and specimens were ground to a thickness of 30 μm. The specimens were stained with Villanueva bone stain.

**Fig. 3:**
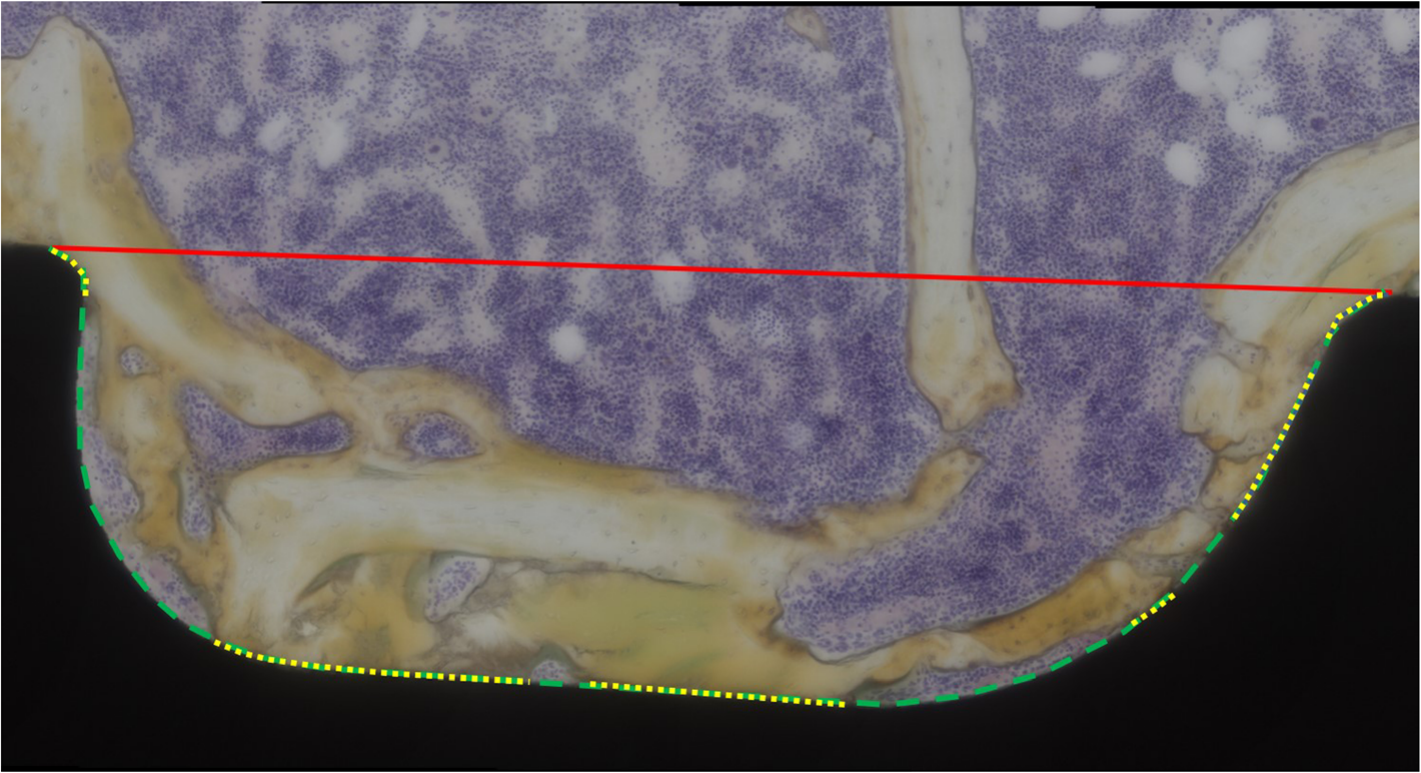
Bone formation index. The ratio of the length of the part where the bone is in direct contact (yellow line) with the length of the inserted screw (green line) was termed bone formation index

**Fig. 4:**
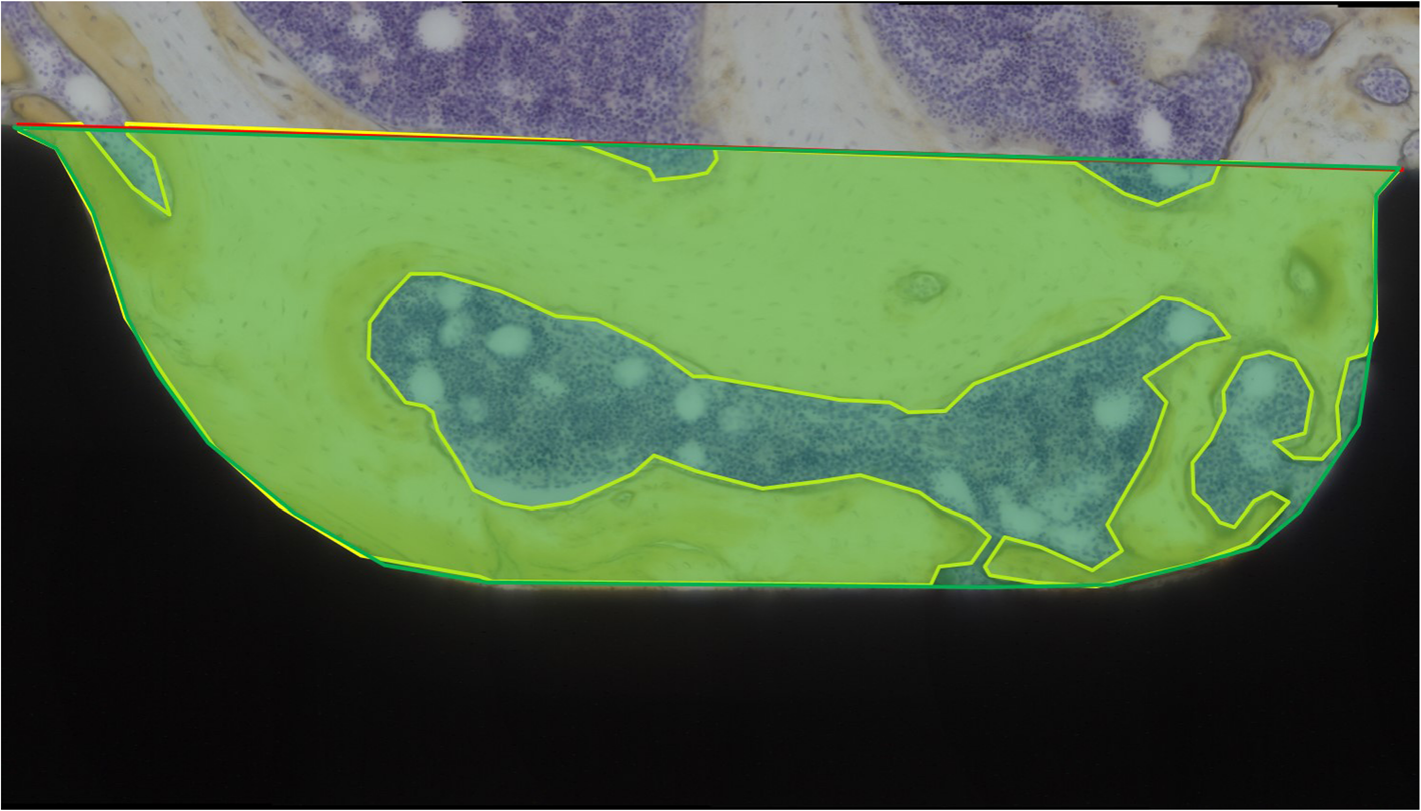
Bone volume density. The ratio of the area of the bone tissue (yellow area) to the area between the threads of the screw (green area) was termed bone volume density

**Fig. 5:**
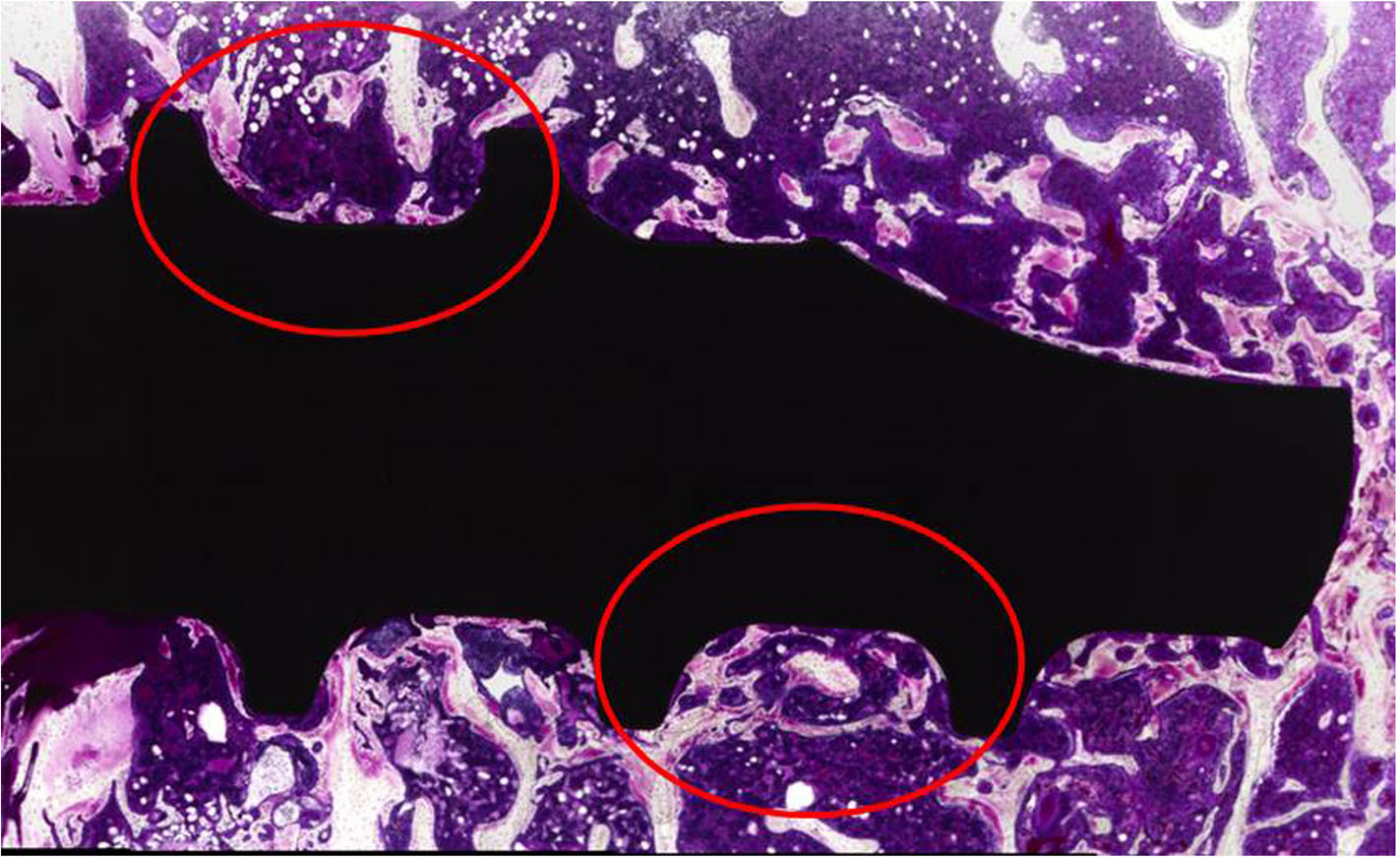
The screw threads used for the histological evaluation. Samples were stained after grinding on the long axis of the screw. The two threads of a screw nearest to the tip were used for histological evaluation (red circles)

Bone formation and bone contact around the screw were evaluated with an optical microscope. According to previous reports, the bone formation index and the bone volume density were analyzed through a histologic evaluation. The bone formation index was defined as the percentage of the length of bone contact on the surface of the screw to the total length of the surface of the screw (Fig. 3) [18]. The bone volume density was defined as the percentage of the area of the bone in the area between the threads of the screw (Fig. 4) [8]. Image J [12] was used for the analysis in this study. The bone formation index and bone volume density of Ti, AO-Ti, and I-Ti screws were compared at 4 and 8 weeks after screw insertion. Two thread points nearest to the tip of the screw in each specimen were also used for the measurement (Fig. 5).

### Statistical analysis

The data were statistically analyzed using the Steel-Dwass test to make the following comparisons: Ti vs. AO-Ti, Ti vs. I-Ti, and AO-Ti vs. I-Ti using the software R version 3.3.0 (Copyright© 2016 The R Foundation for Statistical Computing). The differences were considered significant at the 95% confidence level (*P* < 0.05). After the examinations, the post hoc effect size (Pearson’s correlation coefficient; r) and the actual power of the samples were calculated using G-power software (Franz Faul, Univesitat Kiel, Germany).

## Results

### Biomechanical examination

For the biomechanical test, 12 vertebral bodies (2 dogs) were removed from L1-L6 at 4 and 8 weeks after screw insertion in each group (Ti, AO-TI, and I-Ti). Inappropriate specimens, such as those with poor insertion position of the screw, were excluded. Hence, the number of samples actually adopted for the biomechanical examination was 10 for the Ti and AO-Ti groups and 9 for the I-Ti group at 4 weeks after screw insertion, and 9 for the Ti group and 10 for the AO-Ti and I-Ti groups at 8 weeks after screw insertion.

**Fig. 6:**
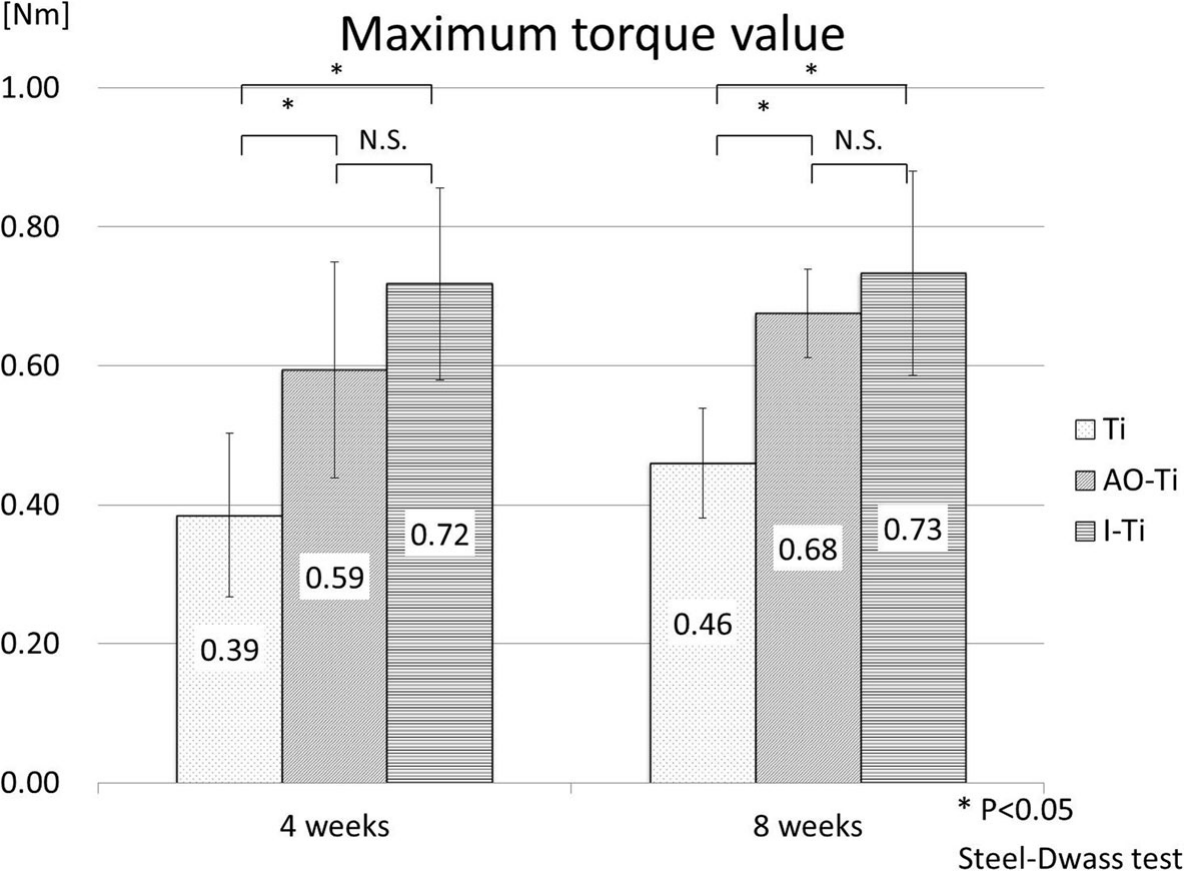
Results of biomechanical examination (maximum torque value). At 4 and 8 weeks after screw insertion, the maximum torque value of the AO-Ti and I-Ti groups was significantly higher than that of the Ti group (*p* < 0.05). There was no significant difference found between the AO-Ti and I-Ti groups

At 4 weeks after screw insertion, the maximum torque value of the AO-Ti group (0.59 ± 0.16 Nm) and I-Ti group (0.72 ± 0.14 Nm) was significantly higher than that of the Ti group (0.39 ± 0.12 Nm) (*p* < 0.05), with the AO-Ti and I-Ti groups showing no significant difference (Steel-Dwass test). At 8 weeks, significant differences were found between the Ti (0.46 ± 0.08 Nm) and AO-Ti (0.68 ± 0.06 Nm) groups, and between the Ti and I-Ti (0.73 ± 0.15 Nm) groups (*p* < 0.05) (Steel-Dwass test) (Fig. 6).

### Histological examination

**Fig. 7:**
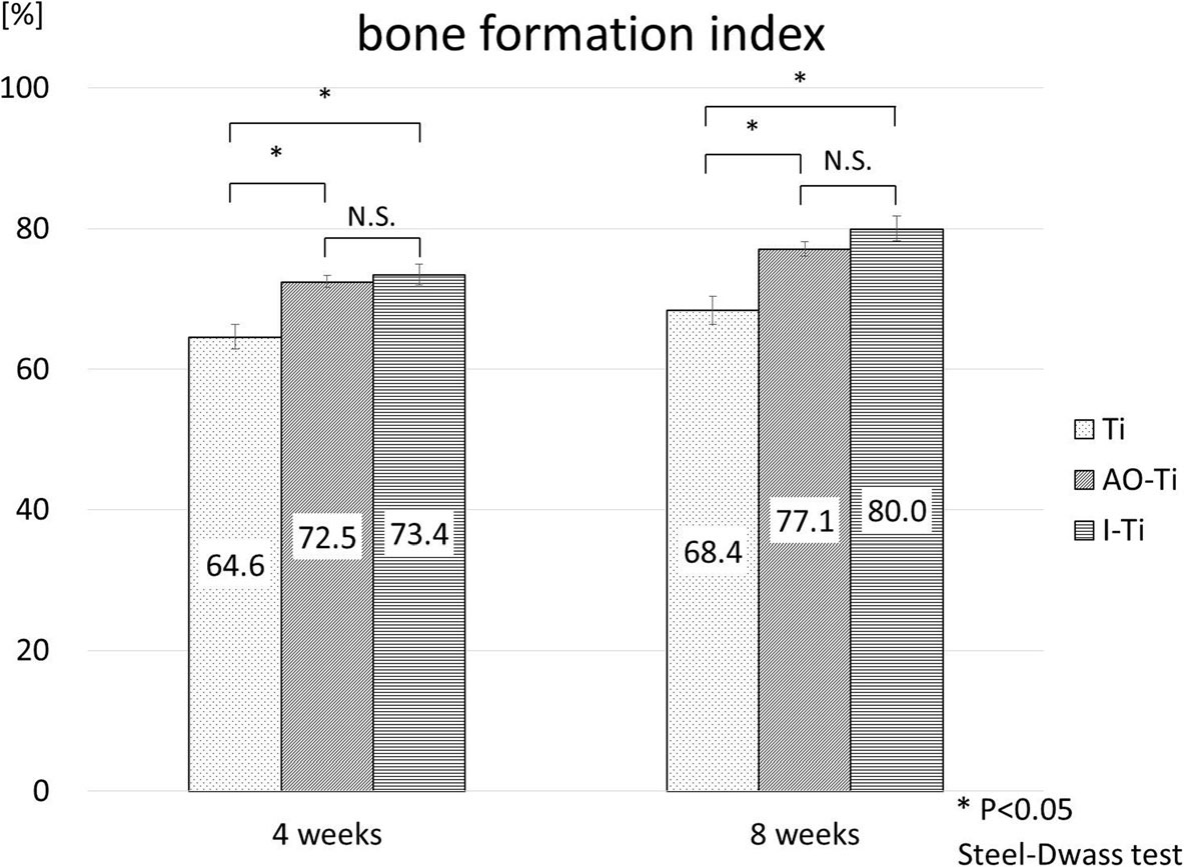
Results of histological examination (bone formation index). At 4 and 8 weeks after screw insertion, bone formation index of the AO-Ti and I-Ti groups was significantly higher than that of the Ti group (p < 0.05). There was no significant difference found between the AO-Ti and I-Ti groups

None of the specimens were excluded in the histological evaluation. Three vertebral bodies were analyzed at 4 and 8 weeks in each group. At 4 weeks after screw insertion, the bone formation index of the AO-Ti (72.5% ± 0.8%) and I-Ti (73.4% ± 1.5%) groups was significantly higher (p < 0.05) than that of the Ti group (64.6% ± 1.7%), with the AO-Ti and I-Ti groups showing no significant difference (Steel-Dwass test). At 8 weeks after screw insertion, the bone formation index of the AO-Ti (77.1% ± 1.0%) and I-Ti (80.0% ± 1.8%) groups was also significantly higher than that of the Ti group (68.4% ± 2.0%), with the AO-Ti and I-Ti groups showing no significant difference (Steel-Dwass test) (Fig. 7).

**Fig. 8:**
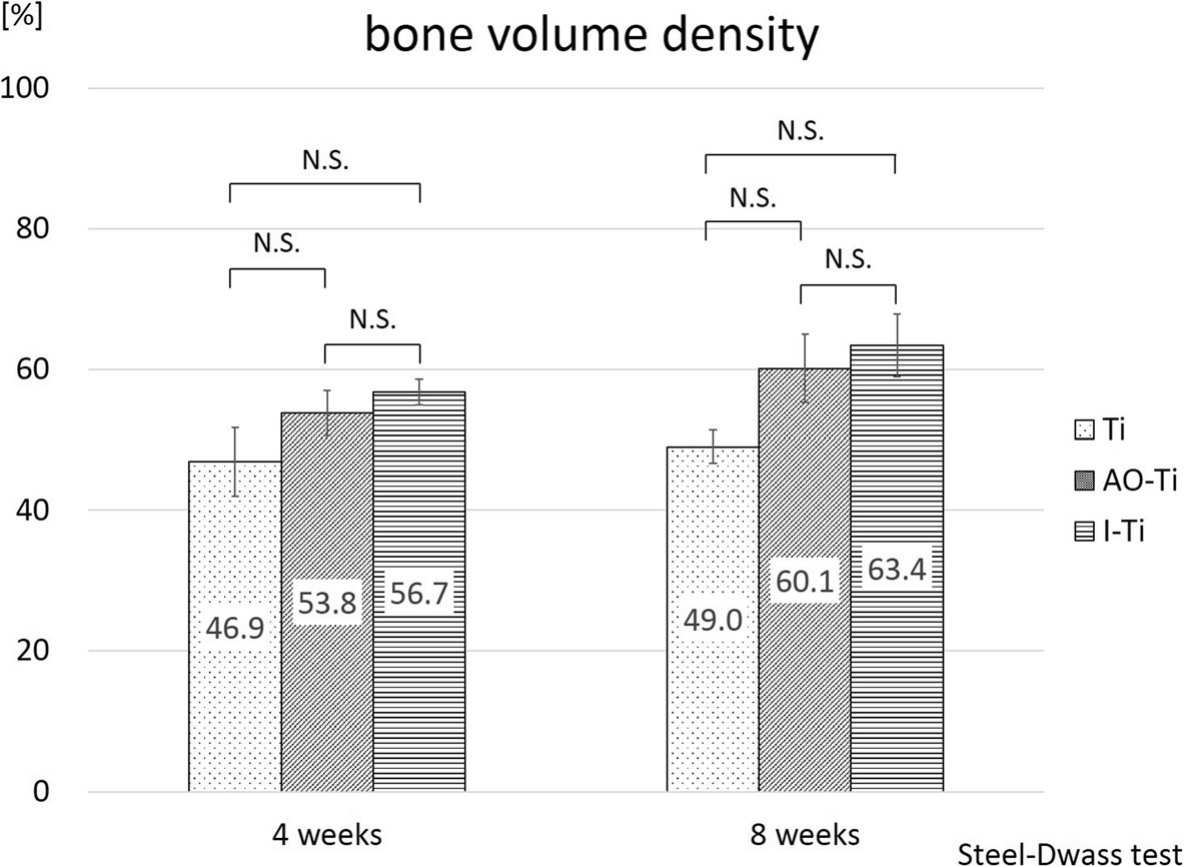
Results of histological examination (bone volume density). The percentage of bone volume density did not show a significant difference between the Ti, AO-Ti, and I-Ti groups at 4 and 8 weeks after screw insertion

The percentage of bone volume density was not significantly different between the Ti, AO-Ti, and I-Ti groups at 4 weeks (Ti group: 64.9% ± 4.9%, AO-Ti group: 53.8% ± 3.2%, I-Ti group: 56.7% ± 1.9%) and 8 weeks (Ti group: 49.0%% ± 2.4%, AO-Ti group: 60.1% ± 4.9%, I-Ti group: 63.4% ± 4.5%) after screw insertion (Steel-Dwass test) (Fig. 8).

## Discussion

In our results, the torque value and the bone formation index of I-Ti were significantly higher than those of Ti and comparable to those of AO-Ti, and the bone volume density showed a similar tendency, although there was no statistically significant difference. This result supports the hypothesis that the osteoconductivity of I-Ti is higher than that of Ti and comparable to that of AO-Ti.

There are several reports that showed the good osteoconductivity of anodically oxidized titanium. Anodically oxidized titanium has a porous and moderately rough structure on the surface, and the surface topology may play an important role in the enhancement of the bone-bonding ability. Liang et al. showed that bone formation was observed on the surface of anodically oxidized titanium directly without intervening the soft tissue [10]. Iwai-Yoshida et al. reported that, on the surface of anodically oxidized titanium, osteogenic gene expressions and nano-biomechanical properties are enhanced [5]. Kim and Ramaswamy reported that the anodically oxidized layer made by anodizing at a high voltage is electrically charged, which also improves the bone reaction and enhances the crystallinity of the oxide [7]. Wang and Li showed that an anodically oxidized titanium surface is covered with a layer of mineral in a simulated body fluid [19]. In our study, AO-Ti and I-Ti had a significantly higher osteoconductivity than Ti, indicating that the anodized layer on the surface of our implant had excellent biocompatibility as reported in previous studies. Previously, Taga et al. showed that I-Ti has a higher pull-out strength than Ti using a cylindrical rod [15]. In our study, we compared the torque values between Ti, AO-Ti, and I-Ti using screws and showed that AO-Ti and I-Ti had higher torque values than Ti. This result suggested that AO-Ti and I-Ti had biomechanical advantages over Ti even when a complex shape, such as a screw, was inserted into the bone.

Iodine-supported titanium was originally developed for the purpose of preventing infection as an antibacterial implant. Shirai et al. cultivated *Staphylococcus aureus* and *Escherichia coli* on stainless steel, untreated titanium, and iodine-supported titanium implants, and showed that both *S. aureus* and *E. coli* formed fewer colonies on the iodine-supported titanium implants than on the stainless steel and untreated titanium implants [13]. Inoue et al. showed that on the surface of the iodine-supported titanium Kirschner wire inserted into the femur of rats inoculated with *S. aureus*, both viable bacteria and biofilm were lesser than those of the untreated titanium and anodized titanium wires at 24, 48, and 72 h after insertion [4]. Although our study did not analyze the antimicrobial activity of I-Ti, we used implants that were made in the same process as that of the previous reports. Therefore, it was considered that the I-Ti implant used in our study had an antibacterial activity equivalent to that shown in the previous reports.

Iodine-supported titanium is made by filling the microporous composite of the anodically oxidized layer with povidone-iodine [3]. Given that iodine-supported titanium has a microporous structure through anodic oxidation on its surface, an excellent osteoconductivity is expected if the encapsulation of povidone-iodine does not have adverse effects.

When using povidone-iodine, toxicity, such as suppression of cell proliferation and povidone iodine-induced burn, is a concern. However, it has been previously shown that iodine-supported titanium has excellent biocompatibility. In basic research, Shirai et al. showed that, when culturing fibroblasts on stainless steel, titanium, and iodine-supported titanium discs, colony formation was not inhibited in either group, and there was a good osteoid formation on the surface of the iodine-supported titanium pin inserted into the femora of the rabbit [13]. Clinically, Tsuchiya et al. reported that none of the iodine-supported implants showed loosening in patients with postoperative infection or compromised status [16]. Kabata showed that good bone formation was observed around the iodine-supported hip prosthesis used for patients with a compromised status or pyogenic arthritis [6]. These reports show that the iodine-titanium implant has a minimum adverse influence in clinical use. Our results also showed that the adverse effects of povidone-iodine were minimal.

Although our findings showed that I-Ti had a higher osteoconductivity than Ti biomechanically and histologically, our study still had some limitations. First, the sample size was small in consideration of the highly invasive surgical procedures. Second, the screws inserted into the vertebral bodies were not connected by rods to eliminate factors, such as loosening due to an external force; thus, they were not suitable for clinical use in spinal surgery. Further investigation is required to determine the biomechanical advantages of I-Ti implants in clinical use with dynamic factors.

## Conclusions

The iodine-supported titanium (I-Ti) implant had a higher osteoconductivity than the titanium implant both biomechanically and histologically. This result indicated that coating anodically oxidized titanium with iodine did not negatively influence the osteoconductivity. In addition to the prevention of surgical site infection, I-Ti can also be expected to decrease the loosening of the screws.

## Data Availability

The datasets used and/or analyzed during the current study are available from the corresponding author on reasonable request.

## Abbreviations

Ti: Untreated Ti-6Al-4 V titanium
AO-Ti: Anodically oxidized Ti-6Al-4 V titanium
I-Ti: Iodine-supported Ti-6Al-4 V titanium

## References

[1] Casarin RC, Casati MZ, Pimentel SP, Cirano FR, Algayer M, Pires PR, Ghiraldini B, Duarte PM, Riberio FV (2014). Resveratrol improves bone repair by modulation of bone morphogenetic proteins and osteopontin gene expression in rats. Int J Oral Maxillofac Surg.

[2] Choi JY, Kim KH, Choy KC, Oh KT, Kim KN (2007). Photocatalytic antibacterial effect of TiO(2) film formed on Ti and TiAg exposed to Lactobacillus acidophilus. J Biomed Mater Res B Appl Biomater.

[3] Hashimoto K, Takaya M, Maejima A, Saruwatari K, Hirata M, Toda Y, Udagawa S (1999). Antimicrobial characteristics of anodic oxidation coating of aluminum impregnated with iodine compound. Inorg Mater.

[4] Inoue D, Kabata T, Ohtani K, Kajino Y, Shirai T, Tsuchiya H (2017). Inhibition of biofilm formation on iodine-supported titanium implants. Int Orthop.

[5] Iwai-Yoshida M, Shibata Y, Wurihan SD, Fujisawa N, Tanimoto Y, Kamijo R, Maki K, Miyazaki T (2012). Antioxidant and osteogenic properties of anodically oxidized titanium. J Mech Behav Biomed Mater.

[6] Kabata T, Maeda T, Kajino Y, Hasegawa K, Inoue D, Yamamoto T, Takagi T, Ohmori T, Tsuchiya H (2015). Iodine-supported hip implants: short term clinical results. Biomed Res Int.

[7] Kim KH, Ramaswamy N (2009). Electrochemical surface modification of titanium in dentistry. Dent Mater J.

[8] Korn P, Schulz MC, Hintze V, Range U, Mai R, Eckelt U, Schnabelrauch M, Möller S, Becher J, Scharnweber D, Stadlinger B (2014). Chondroitin sulfate and sulfated hyaluronan-containing collagen coatings of titanium implants influence peri-implant bone formation in a minipig model. J Biomed Mater Res A.

[9] Lee HJ, Yang IH, Kim SK, Yeo IS, Kwon TK (2015). In vivo comparison between the effects of chemically modified hydrophilic and anodically oxidized titanium surfaces on initial bone healing. J Periodontal Implant Sci.

[10] Liang BJ, Fujibayashi S, Neo M, Tamura J, Kim HM, Uchida M, Kokubo T, Nakamura T (2003). Histological and mechanical investigation of the bone-bonding ability of anodically oxidized titanium in rabbits. Biomaterials.

[11] Nurhaerani AK, Shinonaga Y, Nishino M (2006). Plasma-based fluorine ion implantation into dental materials for inhibition of bacterial adhesion. Dent Mater J.

[12] Schneider CA, Rasband WS, Eliceiri KW (2012). NIH image to ImageJ: 25 years of image analysis. Nat Methods.

[13] Shirai T, Shimizu T, Ohtani K, Zen Y, Takaya M, Tsuchiya H (2011). Antibacterial iodine-supported titanium implants. Acta Biomater.

[14] Shirai T, Tsuchiya H, Shimizu T, Ohtani K, Zen Y, Tomita K (2009). Prevention of pin tract infection with titanium-copper alloys. J Biomed Mater Res B Appl Biomater.

[15] Taga T, Kabata T, Kajino Y, Inoue D, Ohmori T, Yamamoto T, Takagi T, Tsuchiya H (2018). Comparison with the osteoconductivity and bone-bonding ability of the iodine supported titanium with porous oxide layer and the titanium alloy in the rabbit model. J Orthop Sci.

[16] Tsuchiya H, Shirai T, Nishida H, Murakami H, Kabata T, Yamamoto N, Watanabe K, Nakase J (2012). Innovative antimicrobial coating of titanium implants with iodine. J Orthop Sci.

[17] Yang T, Qian S, Qiao Y, Liu X (2016). Cytocompatibility and antibacterial activity of titania nanotubes incorporated with gold nanoparticles. Colloids Surf B Biointerfaces.

[18] Yonekura Y, Miyamoto H, Shimazaki T, Ando Y, Noda I, Mawatari M, Hotokebuchi T (2011). Osteoconductivity of thermal-sprayed silver-containing hydroxyapatite coating in the rat tibia. J Bone Joint Surg (Br).

[19] Wang QQ, Li W, Yang BC (2011). Regulation on the biocompatibility of bioactive titanium metals by type I collagen. J Biomed Mater Res Part A.

